# 436. Paid sick leave among U.S. healthcare personnel, April 2021

**DOI:** 10.1093/ofid/ofac492.511

**Published:** 2022-12-15

**Authors:** Marie de Perio, Anup Srivastav, Hilda Razzaghi, Anthony Laney, Carla Black

**Affiliations:** Centers for Disease Control and Prevention, Cincinnati, Ohio; Leidos Inc, Atlanta, Georgia; CDC, Atlanta, Georgia; National Institute for Occupational Safety and Health, Morgantown, West Virginia; CDC, Atlanta, Georgia

## Abstract

**Background:**

Healthcare personnel (HCP) are at risk for acquiring and transmitting coronavirus disease 2019 (COVID-19), influenza, and other respiratory infections in the workplace. An important strategy to prevent workplace transmission, paid sick leave benefits allow workers to stay home and visit a healthcare provider when ill. Our objectives were to quantify the percentage of HCP reporting paid sick leave, identify differences across occupations and settings, and determine factors associated with having paid sick leave.

**Methods:**

In a national nonprobability Internet panel survey of HCP in April 2021, respondents were asked, “*Does your employer offer paid sick leave?*” We weighted responses to the U.S. HCP population by age, sex, race/ethnicity, work setting, and census region. We calculated the weighted percentage of HCP who reported paid sick leave by occupation, healthcare setting, and type of employment. Using multivariable logistic regression, we identified factors associated with having paid sick leave (i.e. an adjusted prevalence ratio (aPR) with a 95% confidence interval that excludes 1).

**Results:**

In April 2021, 71.4% of 1,652 responding HCP reported having paid sick leave, a slight increase from 68.1% in April 2020. The percentage of HCP reporting paid sick leave varied by occupation, ranging from 54.3% (physicians) to 88.8% (nurse practitioners/physician assistants), and by work setting, ranging from 59.7% (“other” clinical settings) to 82.4% (hospitals). In multivariable analyses, factors associated with having paid sick leave included age between 30–44 years (aPR:1.20) and 45–59 years (aPR:1.22) and male sex (aPR:1.19). HCP working as physicians (aPR:0.74), as contract employees (aPR:0.71), and those working in the Midwest (aPR:0.78) and South (aPR:0.87) regions and in rural settings (aPR:0.86) were less likely to report having paid sick leave.

**Conclusion:**

The majority of HCP from all occupational groups and healthcare settings reported having paid sick leave. However, differences by age, sex, occupation, type of work arrangement, and region exist and highlight disparities. Increasing HCP access to paid sick leave may decrease presenteeism and subsequent transmission of infectious diseases in healthcare settings.

**Disclosures:**

**All Authors**: No reported disclosures.

